# An Analytical Framework for Integrating the Spatiotemporal Dynamics of Environmental Context and Individual Mobility in Exposure Assessment: A Study on the Relationship between Food Environment Exposures and Body Weight

**DOI:** 10.3390/ijerph15092022

**Published:** 2018-09-15

**Authors:** Jue Wang, Mei-Po Kwan

**Affiliations:** Department of Geography and Geographic Information Science, Natural History Building, 1301 W Green Street University of Illinois at Urbana-Champaign, Urbana, IL 61801, USA; mpk654@gmail.com

**Keywords:** environmental health, food environment, environmental context cube, environmental context exposure index, the uncertain geographic context problem (UGCoP), GPS, GIS

## Abstract

In past studies, individual environmental exposures were largely measured in a static manner. In this study, we develop and implement an analytical framework that dynamically represents environmental context (the environmental context cube) and effectively integrates individual daily movement (individual space-time tunnel) for accurately deriving individual environmental exposures (the environmental context exposure index). The framework is applied to examine the relationship between food environment exposures and the overweight status of 46 participants using data collected with global positioning systems (GPS) in Columbus, Ohio, and binary logistic regression models. The results indicate that the proposed framework generates more reliable measurements of individual food environment exposures when compared to other widely used methods. Taking into account the complex spatial and temporal dynamics of individual environmental exposures, the proposed framework also helps to mitigate the uncertain geographic context problem (UGCoP). It can be used in other environmental health studies concerning environmental influences on a wide range of health behaviors and outcomes.

## 1. Instruction

Environment-related chronic diseases are one of the biggest threats to public health. According to the World Health Organization (WHO), 22% of the global burden of disease is caused by environmental risks [1]. Because environmental exposure is a significant factor that influences health behaviors and outcomes, researchers in public health and health geography have put considerable effort into assessing environmental impacts on health [2,3,4]. Evidence shows that exposures to different environmental factors, such as air and noise pollution [5,6,7], the built environment [2,4,8,9] and the food environment [10,11,12,13,14], have significant associations with various health behaviors, including physical activity [2,15,16], tobacco and drug use [17,18,19,20], and health outcomes, which includes obesity and obesity-related disease [21,22,23,24,25] and mental health disorders [26,27,28,29].

The food environment, among different environmental factors, is one of the critical factors that could lead to obesity [30] and other obesity-related chronic diseases such as type II diabetes [31] and cardiovascular diseases [32], whose prevalence has increased rapidly in recent decades [33]. Increasing public concerns have prompted a growing number of studies on the effects of food environment exposures on obesity. Previous studies found that living in food desserts [34] and exposures to unhealthy food outlets [35] may encourage unhealthy food intake behavior that is associated with a higher likelihood of obesity. Furthermore, the association between food environment exposures and obesity has been utilized in developing intervention strategies to improve public health by numerous institutions worldwide [36,37,38]. 

However, the findings of the effects of food environment exposures on obesity are inconsistent [13,39,40]. Although a higher likelihood of obesity has been found to be significantly associated with exposures to unhealthy food (e.g., fast food restaurants) in many studies [12,41], it was not observed in other research [42,43,44]. For example, exposures to fast food restaurants were found to be positively associated with the prevalence of obesity in some studies [35,45,46], while no correlation [47,48] or even negative association [49] was observed in other studies. The inconsistent findings regarding the effects of the food environment on obesity bring enormous challenges on implementing effective policy to improve public health. 

Many potential issues could cause these inconsistent findings (e.g., the modifiable areal unit problem [50], the uncertain geographic context problem [50], and spatial non-stationarity [51]). To examine whether the food environment has significant influences on obesity, an important task is to accurately measure individual exposure to relevant environmental factors [52]. Past studies that examine the effects of environmental exposures on health outcomes have predominantly used residential neighborhoods as contextual units [53]. In these studies, residential neighborhoods were defined either by the administration units (e.g., census tracts) in which people’s homes are located or by buffer areas with a specific radius around people’s home location [54,55,56]. However, residential neighborhoods only partially represent the environmental context that affects people’s health, since people move around in their daily life [57,58,59]. Identifying environmental context based solely on the residential neighborhood may thus lead to inaccurate contextual exposure assessment and erroneous results concerning the relationships between environmental contexts and health outcomes [60,61]. This methodological issue may contribute to the inconsistent findings of past studies and has been articulated as the uncertain geographic context problem (UGCoP) by Kwan [62]. 

The UGCoP refers to the problem that findings about the effects of environmental factors (e.g., exposure to fast food restaurants) on individual health outcomes (e.g., obesity) could be affected by how contextual units are geographically delineated and the “temporal uncertainty in the timing and duration in which individuals experienced these contextual influences” [63]. In light of the dynamic nature of people’s daily activity, people’s movement in space and time should be taken into account while measuring their food environment exposures and its effects on obesity [60]. To mitigate the UGCoP, portable GPS devices can be utilized to accurately trace human movement in space and time, and advanced GIS methods can be used to relate activity locations to relevant environmental risk factors [63,64]. Further, GPS trajectories can be used to derive human activity space, which is more representative of people’s daily context than the residential neighborhood [65]. The integration of GPS and GIS provides a powerful means for investigating the relationships between environmental contexts and health outcomes [58,66]. 

Individual GPS trajectories capture people’s movement in space and time and thus help mitigate the UGCoP through more accurately delineating contextual units. A growing number of studies have started to adopt GPS-based activity space methods to investigate environmental effects on health outcomes [30,67,68]. However, environmental contexts can vary over time in a highly complex manner and are thus temporally uncertain [50]. Some contextual variables change over the 24-h period of a day (e.g., air pollution and the food environment), and some change over the seasons [7,69]. The temporal uncertainty of the environmental context is mostly ignored and not taken into account in environmental exposure assessment in previous studies. With regard to the food environment, food outlets open and close according to their daily schedules. Furthermore, many food outlets have different opening hours for weekdays and weekends. Given the complexity and spatiotemporal uncertainties of the food environment, it is highly challenging to accurately delineate the environmental context and assess individual exposures to the food environment. The UGCoP may introduce considerable errors to the results if the spatial and temporal variability of the food environment and people’s daily movement in relation to such environment are not appropriately considered, but most previous research has paid little attention to them.

To address the challenges of the UGCoP, this paper proposes an analytical framework that utilizes GIS, time geography and GPS trajectory data to assess individual food environmental exposures for environmental health studies. In the framework, an environmental context cube (ECC) integrates variations in the food environment over space and time into a 3-D cube. By buffering individual GPS trajectories in 3-D to generate the individual space-time tunnel (ISTT) and projecting it into the ECC, environmental exposures can be derived and assessed by identifying the 3-D intersection of the ISTT and ECC. Based on the intersection, we calculate the environmental context exposure index (ECEI) as a standardized measure of individual exposure to the food environment. Considering both the spatiotemporal variations in the food environment and the dynamics of people’s daily movement, the ECEI may provide a more accurate and reliable measurement of individual exposure to the food environment. The ECEI is utilized in this study to explore the relationship between individual food environment exposures and the overweight status for 46 participants using data collected with GPS in Columbus, Ohio, and binary logistic regression models. The results indicate that the proposed framework is effective for assessing individual exposures and investigating their health effects when compared with other widely used food environment exposures assessment methods. Addressing the spatiotemporal variations of contextual influences, the framework may help mitigate the spatial and temporal uncertainties in the food environment in public health studies. Further, the methodology is also useful in a wide range of environmental health research.

## 2. The Proposed Analytical Framework 

**Figure 1:**
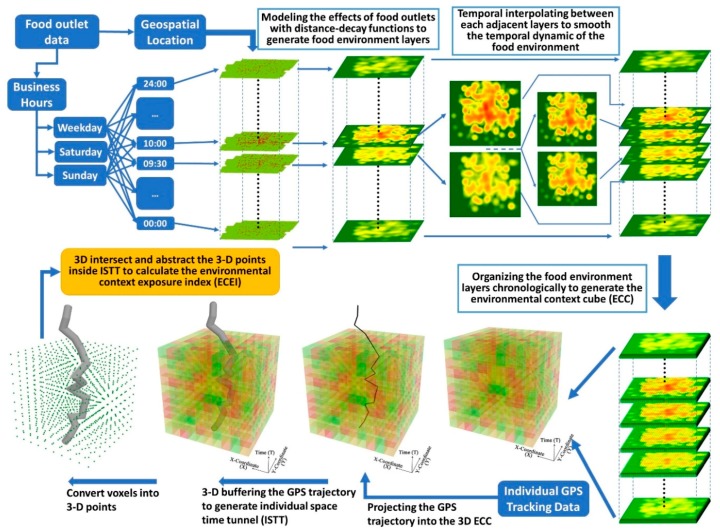
The proposed analytical framework.

This study proposes an analytical framework for assessing the effects of environmental exposures on individual health outcomes using food environment as an example. The framework seeks to more accurately measure individual food environment exposures so that more robust research findings can be obtained. Figure 1 illustrates the framework that integrates GIS, time geography, and GPS tracking. For this study, the food environment (BMI-unhealthy food outlets) is selected to generate 3-D environmental context cubes (ECC) for weekdays, Saturdays, and Sundays, which represent the spatial and temporal dynamics of the environmental contexts. By buffering individual GPS trajectories in 3-D, an individual space-time tunnel (ISTT) is generated to represent individual exposure space. By projecting the 3-D ISTT into the corresponding 3-D ECC and identifying their intersection, the individual environmental context exposure index (ECEI) can be derived as a standardized exposure measure. Based on the ECEI, the effect of the food environment on overweight is explored with statistical models, and the results are compared with other methods. Details of the dataset, the ECC, the ISTT and the ECEI are discussed in the following sections.

### 2.1. Study Area and Data

The study area for this research is Franklin County (Ohio, U.S.), which is part of the Columbus metropolitan area and where the city of Columbus is located. It is the second-most-populated county in Ohio where the percentage of obese or overweight adults is 63.9% [70]. The county includes urban, suburban, and some rural areas. This characteristic is helpful for a study that seeks to consider the influences of various land uses on people’s health outcomes. In addition, Franklin County has a diverse racial composition, and also has both wealthy and impoverished communities. Further, there are 3727 food outlets in the county that include many kinds of food retailers (e.g., fast food restaurants, full-service restaurants, and supermarkets) that provide different types of foods. These features of Franklin County facilitate the identification of the spatial heterogeneity of the food environment and the differences in the levels of exposure among the county’s population with various socio-economic statuses.

The GPS trajectory dataset used in this research was collected as part of a larger study that examines the influence of parks on people’s physical activity in four U.S. cities: Albuquerque (NM), Chapel Hill and Durham (NC), Columbus (OH), and Philadelphia (PA). In each of these cities, participants were recruited in person in selected public parks and neighborhoods surrounding these parks following household interviews. For each selected park and its surrounding neighborhoods, about 300 persons from different socio-economic backgrounds were randomly solicited to participate in the study. In the end, 238 subjects participated in the study and 51 of them were from the Columbus study site. Participants in the study were asked to wear a GPS and an accelerometer for three consecutive weeks. The data were collected from August 2009 to October 2010 in three selected seasons (spring, summer, and fall) to avoid the winter months in which people may undertake fewer outdoor physical activities (since the purpose of the larger project was to assess the influence of parks on people’s physical activity). Geographic location was recorded by the GPS devices with a time interval of one minute. In addition, data about subjects’ demographic, anthropometric and socio-economic statuses were also collected. Subjects’ overweight status in the dataset was assessed by the Body Mass Index (BMI), which was calculated by dividing the subject’s weight (kg) with his or her height in meters squared (m^2^). 

The environmental context data for this study were derived from a comprehensive digital geographic database of Franklin County maintained by the Franklin County Auditor’s Office. It includes the attributes and physical boundaries of relevant environmental contexts. Food outlets data were derived from the food license data of Franklin County, and their business hours were collected and confirmed using Google Map and phone calls. These data include each food outlet’s business name, geographic location, business hours and business category according to standard industrial classifications.

### 2.2. Data Pre-Processing

GPS signals may be absent in locations near tall buildings or under dense tree canopies, and this may lead to gaps in GPS tracking data. Data pre-processing is thus necessary to improve the reliability and usefulness of GPS data. Consistent with the procedures used by Wiehe et al. [71], missing GPS records in the Columbus GPS dataset were inserted at the location of the earlier point if the distance between two temporally adjacent records bounding a period of missing data was less than 30 m. If this distance was longer than 30 m and the gap between two recorded GPS points was less than 1 h, interim 1-min time points were imputed. Missing GPS points for time periods longer than an hour were considered missing and not imputed. Further, only survey days with eight or more hours of valid GPS records for each subject were included in the analysis.

Although the original dataset had 51 participants, only subjects with valid GPS records for at least five weekdays and two weekend days were included in the analysis. This ensured that there were sufficient data for the selected subjects for at least seven days that covered their daily activities in both weekdays and weekend days. As a result, 46 participants were finally selected as valid subjects for further analysis. Although the sample size is not large, the subjects cover a range of socio-economic attributes (e.g., age and education level) and are thus useful for this exploratory study of the proposed analytical framework for food environment exposure measurement. 

Table 1 shows the demographic and socio-economic characteristics of the 46 participants in the sample used in the study. These participants are predominately female (60.87%) and younger people. All of them are adults, and only 2.18% are seniors older than 65. With respect to the education level, 56.52% of the subjects have a college degree or higher, while 43.58% of them have a high school degree or lower. The overweight status among the 46 participants is balanced in that half of them are overweight and the other half are not overweight.

### 2.3. Representing Dynamic Environmental Contexts Using the Environmental Context Cube

Any attempt to measure exposures to the food environment needs to begin with representing the food environment, which in turn requires researchers to consider the location and distribution of food outlets as well as the kinds of food those outlets provide. The geographic range of influence from food outlets can be assessed by creating homogeneous buffer areas covering food outlet locations with a specific distance (such as 100 m or 1 km). Importantly, however, representation of food outlets’ effects on health behavior should take into account the effect of distance decay rather than using arbitrary distance cutoffs: Environmental effects change as a function of distance, with locations farther from a food outlet less influenced by that outlet than nearer locations are. A few researchers have included distance-decay functions [30,72,73,74,75,76,77] as part of their food environment studies with a view to accounting for the effect of distance, but even these studies have treated the food environment statically and have failed to consider the dynamic features of the food environment (e.g., food outlets’ opening and closing at different times of the day).

As some researchers have noted [39,62], environmental contexts undergo continuous change. For example, air pollution levels differ throughout the day. For this reason, exposure assessment may produce erroneous results if the variability of the environmental context is ignored [7]. Furthermore, the contextual influences of the food environment may also differ with time of day. Previous studies have largely ignored temporal variations in the food environment, although most food outlets operate on specific schedules and offer their services only during certain hours. Some even feature different schedules for weekdays and weekends. Accordingly, Chen and Clark [78], arguing that conventional space-only methods of exposure assessment overlook the dynamic features of the food environment, proposed a spatiotemporal method that takes into account food outlets’ business hours when measuring access to food retailers. Although portrayal of the food environment spatiotemporally in Chen and Clark’s [78] study is an important step forward for the study of environmental health, the study measures food access using census tracts as its contextual units and thus may still be susceptible to the UGCoP. As a result, further development is need to more accurately assess individual exposures to the food environment and to mitigate the UGCoP. 

**Figure A1:**
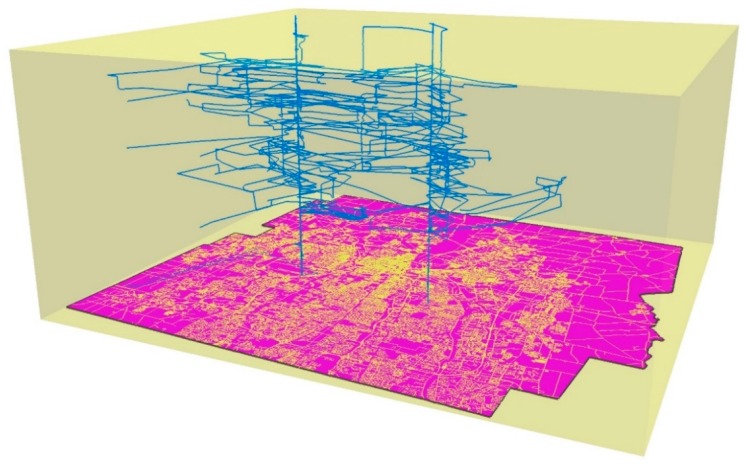
An example of the space-time cube (aquarium).

The ECC is developed in this study to address both spatial and temporal uncertainties in the food environment while accurately assessing individual exposures to that environment. It is designed to capture the complex dynamics of environmental contexts as well as individual exposures. Indeed, this extension of the space-time cube [79] is explicitly designed for use in environmental health research. The base of the space-time cube (or space-time aquarium), which is a time-geographic construct first introduced by Hägerstrand in the 1960s, represents the geographic contexts of the study area (*x*-axis and *y*-axis), with 3-D lines inside the cube representing an individual’s movement trajectories. The cube’s vertical dimension (*z*-axis) represents time. Figure A1 in the Appendix A illustrates a space-time cube that integrates geographic contexts and GPS trajectories. Note, however, that this representation visualizes only individual movement trajectories in 3-D, whereas the environmental context is represented on a 2-D plane. Constrained by the 2-D plane, the environmental context can be visualized and analyzed at only one time point using this space-time cube framework. In real-world contexts, however, environmental contexts and their influence on moving subjects may change over both space and time in highly complex ways. Representation of the environmental context should thus be also extended to capture and represent the dynamic features of the environment by integrating time as the third dimension. 

By extending the traditional space-time cube, we propose the environmental context cube (ECC) as a new analytical framework for analyzing people’s movement and their dynamic relationships with their environmental context (e.g., the food environment). The ECC is a collection of 3-D voxels arranged on a regular grid in 3-D space. The value of each voxel represents the environmental context at a specific geographic location (*x*- and *y*-coordinate) at a specific time (*z*-coordinate). Thus, spatial and temporal variations in the environmental context are rendered as the different values of the voxels in 3-D space at various locations and times. In the temporal dimension of the cube, layers of voxels constitute the ECC, with each layer representing the spatial configuration of the environmental context at a specific time of day. The size of the voxels in the 3-D space represents the spatial and temporal resolutions: The higher the spatial resolution, the more detailed the spatial variations represented; and the finer the temporal resolution, the more detailed is the representation of the temporal dynamics. Different spatiotemporal resolutions of the ECC may affect the accuracy with which variations in the environmental context can be captured. To thoroughly examine the ECC while exploring the effect of spatiotemporal resolutions (or scale effects) on measurement accuracy, we built ECCs featuring combinations of three different spatial resolutions (100 m × 100 m, 150 m × 150 m, 200 m × 200 m) and two temporal resolutions (30 min, 10 min), and then evaluated and compared their performance.

**Figure 2:**
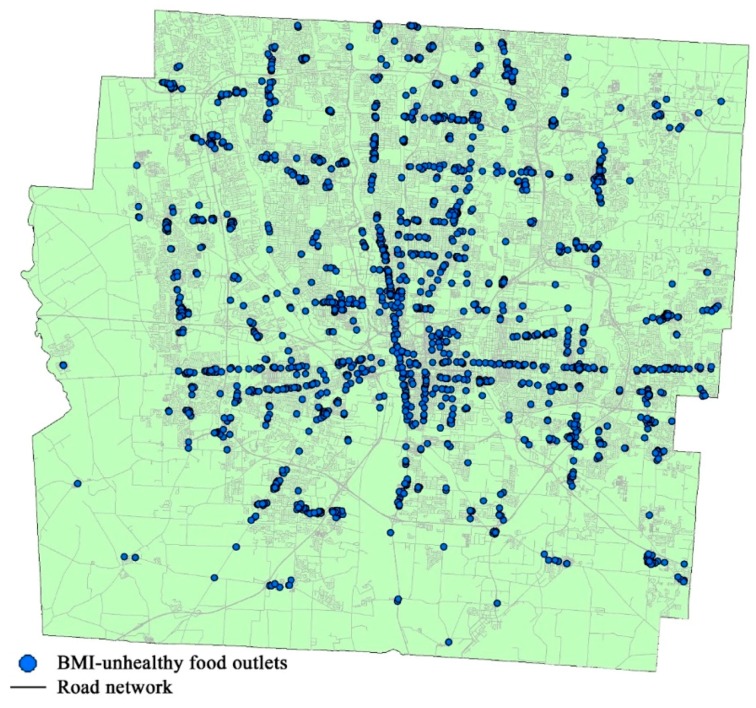
The study area (Franklin County) and the location of BMI-unhealthy food outlets.

In this manner, we constructed a series of 3-D ECCs to represent the unhealthy food environment of the study area. We classified the food outlets in the study area into three categories as described by Rundle et al. [80]—BMI-healthy, BMI-unhealthy, BMI-neutral. Because exposure to BMI-unhealthy food outlets may be related to high BMI, as has been observed in many previous studies [41,45,46,81], BMI-unhealthy food outlets were used in this study to generate 3-D ECCs for measuring individual exposure to unhealthy food environment. In Rundle et al.’s [80] classification system, BMI-unhealthy food outlets include fast-food restaurants, convenience stores, meat markets, pizzerias, bakeries, and candy and nut stores. Franklin County, the study area, contains 1645 BMI-unhealthy food outlets. All of them are included in the food environment analysis in this study. Figure 2 shows the location of these BMI-unhealthy food outlets.

In analyzing the business hours of the 1645 BMI-unhealthy food outlets included in the study, we found that many food outlets had different schedules on weekdays and weekends; some even had different schedules on Saturdays and Sundays. To give just one example, a restaurant might be open from 10 a.m. to 10 p.m. on weekdays, from 11 a.m. to 10 p.m. on Saturdays, and from 11 a.m. to 8 p.m. on Sundays. Accordingly, we generated three different environmental context cubes (ECCs) to better represent this dynamic food environment: one for weekdays, one for Saturdays, and the other for Sundays. To construct the ECC for one day, we first generated layers of the food environment at different times of the day. For ECCs with a temporal resolution of 30 min, we generated a food environment layer for each of the 48 half-hour time slots in the day. For each time slot, a food environment layer was created to estimate the extent and degree of environmental effects of BMI-unhealthy food outlets based on the locations of the outlets open at that specific time. 

**Figure 3:**
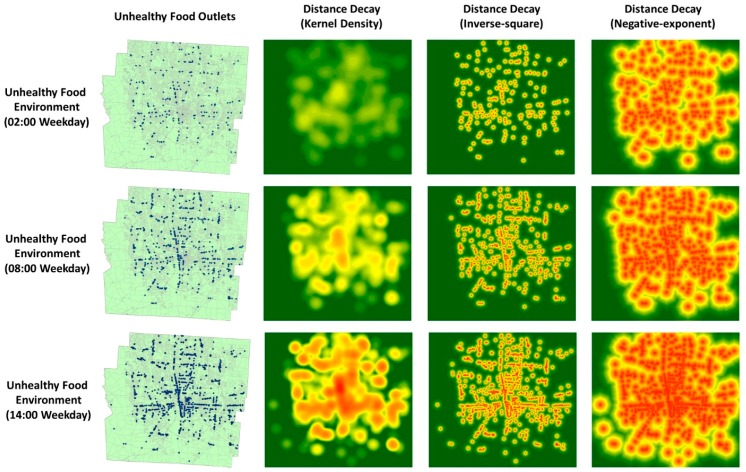
The food environment layers at different time of a day generated by the three different distance-decay methods (the food environment layers with spatial resolution of 100 m × 100 m are used in this figure to illustrate the three distance-decay methods).

As already noted, any assessment of the environmental influence of food outlets should take into account the effects of distance decay. For this reason, based on the location of the food outlets operating during each of the 48 time slots of a day, we modeled the decline in each food outlet’s influence using three distance-decay methods that were used in previous environmental health studies [30,72,73,74,75,76,77]: kernel density estimation (KD), an inverse-square distance-decay function (ISDD), and a negative-exponential distance-decay function (NEDD). The food environment at a specific time of day (a time slot) was thus represented as a raster layer created by estimating the extent and degree of the environmental effects of BMI-unhealthy food outlets based on the locations of food outlets operating at that time of day, using one of the three distance-decay functions. Figure 3 shows the food environment layers at three different time slots of the same day, as generated by the three distance-decay methods. Separate food environment layers were generated for the 48 time slots using each of these methods for three kinds of days (weekdays, Saturdays and Sundays). Then, for each kind of day, the 48 food environment raster layers were voxelized with the unit size of 30 min and mapped to the *z*-axis. In this way, a 3-D environmental context cube (ECC) was constructed by organizing the 48 voxelized layers chronologically using the assigned z-values (which represent the specific time corresponding to each layer). Because the ECCs were implemented using three distance-decay methods (KD, ISDD, NEDD) in three spatial resolutions (100 m × 100 m, 150 m × 150 m, 200 m × 200 m) for three kinds of day (weekdays, Saturdays, and Sundays), 27 ECCs were ultimately constructed with a temporal resolution of 30 min.

**Figure 4:**
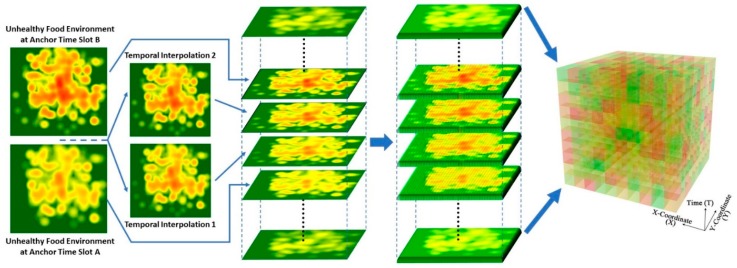
The process of the temporal interpolation and the generation of the 3-D environmental context cube with food environment layers.

To capture more details of the temporal variations in the food environment, another 27 environmental context cubes (ECCs) with a temporal resolution of 10 min were constructed using the same three distance-decay methods, three spatial resolutions, and three kinds of day. Each of these ECCs has 144 food environment layers, where each layer represents each of the 10-min slots that make up the 24 h of a day (e.g., 10:10 a.m., 10:20 a.m. and so on). These layers were calculated by raster algebra using linear interpolation, in which the pixel value of an additional layer (in addition to the original 48 layers at the resolution of 30 min) was calculated using linear polynomials based on the values of the corresponding pixels in the two temporally adjacent layers among the 48 30-min resolution layers. These 144 food environment layers were then voxelized, mapped to the *z*-axis, and organized chronologically to form the ECCs with a temporal resolution of 10 min. This higher temporal resolution allowed the temporal dynamics of the food environment in any particular day to be better represented. Figure 4 illustrates the process of the temporal interpolation as well as the generation of a 3-D ECC and its food environment layers.

**Figure 5:**
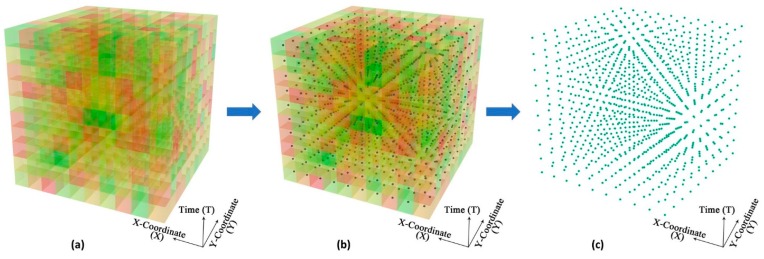
Implementation of the 3-D environmental context cube using a 3-D point cloud. (**a**): an environmental context cube, (**b**) voxels in the cube represented by points at the centroid of the original voxels, (**c**) the corresponding 3-D point cloud.

To facilitate the implementation and computation of the 3-D environmental context cubes (ECCs), we converted each 3-D ECC to a 3-D point cloud, with each voxel in the cube represented by a point in the cloud at the centroid of the original voxel. As shown in Figure 5, the three dimensions of the points correspond to the *x*-coordinate (X), *y*-coordinate (Y), and time (T) seen in the original ECC. The values of the environmental factors were stored as an attribute table linked to each point in the 3-D point cloud. 

### 2.4. Capturing the Spatiotemporal Exposure Space with Individual Space-Time Tunnel

Using GPS trajectory data for individual exposure assessment could capture the daily movement of people and thus help mitigate the UGCoP to some degree. However, conventional methods capture only the spatial extents of individuals’ activities using 2-D polygons that represent a person’s activity space (e.g., GPS trajectory buffers, standard deviation ellipses, and minimum convex polygons) or, at most, weigh the accumulated time spent at different activity locations (e.g., kernel density surface, context-based crystal-growth activity space [68]), where environmental contexts were considered statically and the dynamics of the food environment ignored. Although these methods consider the accumulated time that an individual spends at different locations (e.g., person A spends 8 h at the workplace on weekdays), they ignore temporal variations in people’s location of activity (e.g., person A stays at the workplace from 8 a.m. to 12 p.m. and from 1 p.m. to 5 p.m. on weekdays). Knowledge of the exact times when a person is at a location is essential for understanding the resulting level of exposure to the food environment. For example, consider a person who visits an area where many fast food restaurants may be found but does so at 1 a.m., when they are closed. Conventional activity space methods would include this occurrence in the environmental context exposure assessment even though the person was not actually exposed to fast food restaurants at that time. In this way, overlooking temporal variations in the food context and the exact times when people undertake their daily activities at various locations may introduce measurement error.

**Figure 6:**
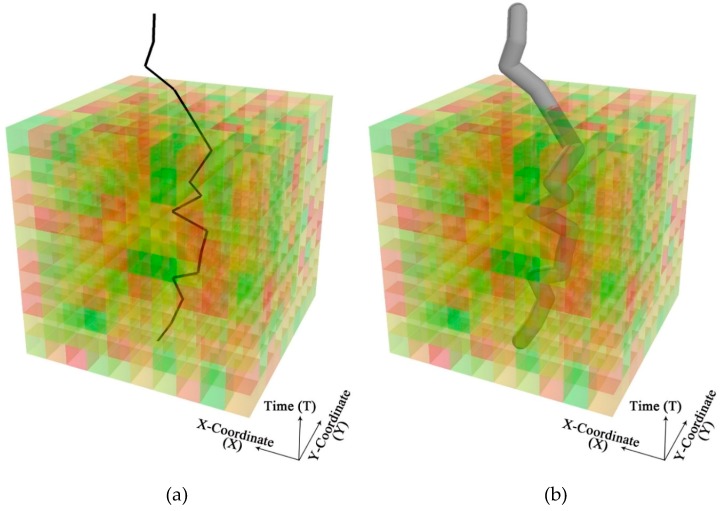
A GPS trajectory (**a**) and an individual space-time tunnel (**b**) projected into an environmental context cube.

To help address the temporal uncertainty that is an essential element of the environmental context and the dynamics of people’s daily activity, we propose the individual space-time tunnel (ISTT) as a way of representing the individual exposure space. The ISTT was generated by a 3-D buffer of an individual’s GPS trajectory at a specific distance (e.g., 100 m) in a 3-D space. To generate the ISTT, GPS trajectories were projected into the ECC according to the geographic coordinates and timestamps of the GPS records. As shown in Figure 6a, the GPS trajectory of a subject is projected into the 3-D space of an ECC, much like the space-time paths inside a space-time aquarium. The voxels along a particular trajectory and its surrounding areas constitute the environmental context that influences the corresponding subject. Thus, environmental exposure should be derived using a 3-D buffer space of appropriate radius around people’s movement trajectories, as shown in Figure 6b. The buffer radius $B_{r}$, a user-defined parameter that represents the effective range of a particular environmental influence, can vary for different population groups based on individual socio-demographic attributes (e.g., age). For example, older adults and children may have a smaller $B_{r}$ than adolescents do, because they both tend to have lower mobility. We might also define $B_{r}$ as a function of travel velocity. For example, we might associate higher velocity with smaller $B_{r}$, noting that higher velocity (i.e., quicker bypass) may allow for less influence on the subject from the environmental context around a location. For purpose of illustration, we set $B_{r}$ to 100 m in this exploratory study.

### 2.5. Measuring Food Environment Exposure with the Environmental Context Exposure Index

**Figure 7:**
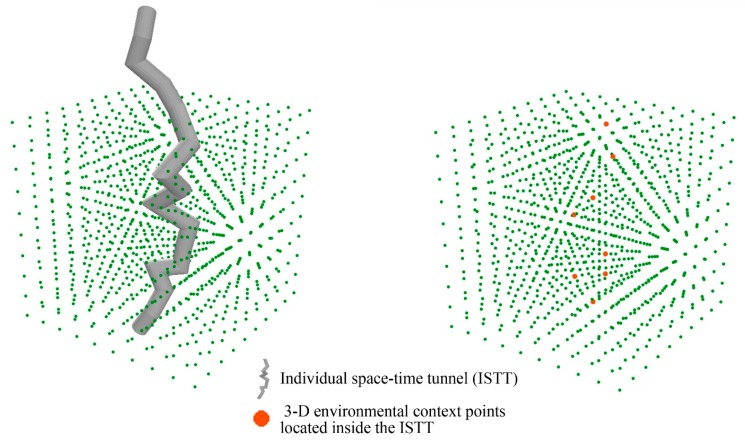
The 3-D intersection of the point cloud and individual space-time tunnel.

The proposed 3-D environmental context cube (ECC) can capture the complexity and dynamics of the food environment, and the individual space-time tunnel (ISTT) can delineate the individual spatiotemporal exposure space by integrating spatial as well as temporal variations in a person’s daily activities. As a result, individual exposures to the food environment can be derived by the 3-D intersection of the ECC and the ISTT: By projecting the 3-D ISTT into the corresponding point cloud of the ECC, as illustrated in Figure 7, we can link exposure to the food environment with all points located inside the ISTT in the 3-D space. The results of the 3-D intersection allow calculation of the environmental context exposure index (ECEI), which in turn allows the measurement of individual exposures to the environmental context. By capturing the extent to which a person is exposed to the relevant environmental context during each time unit throughout a day, the ECEI provides a new method for quantifying an individual’s level of exposures to the food environment. In this study, the ECEI was used to analyze the association between individual exposure to the food environment and health outcomes.

By identifying the 3-D intersection of the ISTT and the point cloud, subjects’ exposures to the food environment can then be derived. After abstracting all intersected 3-D points from the ECC, the ECEI was evaluated as follows:

(1) $$ECEI\left(j\right)\left(k\right)=\;\sum_{i=1}^{n}\frac{EC_{ij}W_{i}}{T}\;\;\;\;\left(1\leq i\leq n\right)$$

(2) $$W_{i}=\{\begin{array}{cc} 1 & \;\;if\;v_{i}=0\\ {\left(\frac{1}{2}\right)}^{v_{i}} & \;\;if\;v_{i}>0 \end{array}\;\;\;(1\leq i\leq n)$$

where ECEI(j)(k) is the environmental context exposure index of environmental factor j for subject k, $EC_{ij}$ is the value of environmental factor j for 3-D point i based on the intersection of the ISTT and the ECC, $W_{i}$ is the weight for 3-D point i, T is the time span of the intersection (the time unit can be hour or day), and n is the total number of points derived by the 3-D intersection.

$W_{i}$ is a user-defined parameter for calculating the environmental context exposure index (ECEI). In most cases, the point weight ($W_{i}$) can be set to 1, but it can also differ for different research questions. One possible method for assigning voxel weight is to use movement velocity, as shown in Equation (2), where $v_{i}$ is the movement velocity of the subject when passing through voxel i: The higher the velocity, the smaller the weight should be, indicating less contextual influences. When speed equals 0, the subject is staying at that location, so weight is set to 1. A quickly moving subject, by contrast, may pass by a location quite rapidly; thus, the influence of the environmental factor at 3-D point i should be small and the weight less than 1. Again, for purpose of illustration, $W_{i}$ was set to 1 globally for this exploratory study.

Using this method, we calculated an environmental context exposure index (ECEI) for each of the 54 environmental context cubes (ECCs) separately. Because the three distance-decay methods generate ECCs with different value ranges, we standardized the ECEI by using z-score to facilitate the comparison of the exposures measured by different ECCs. 

### 2.6. Comparing the Individual Food Environment Exposure Measurement with Other Methods

**Figure 8:**
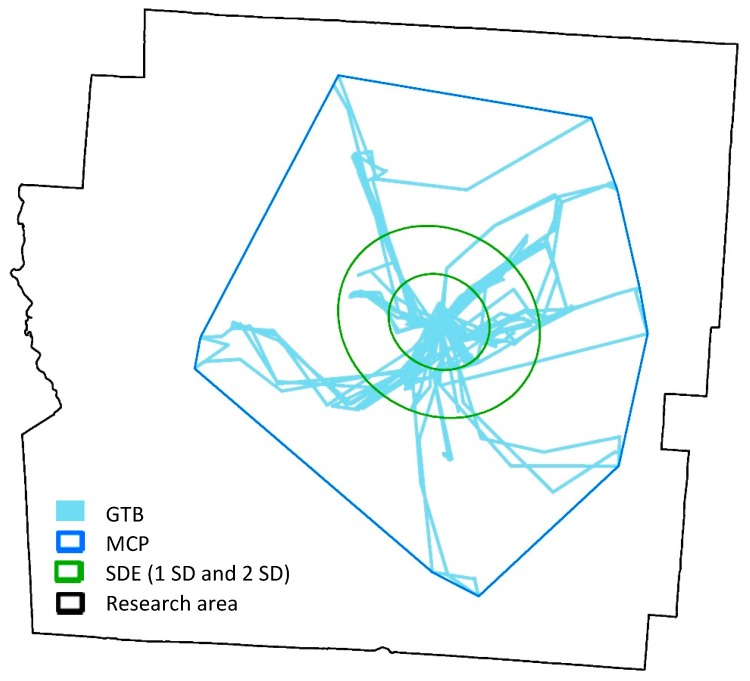
Four widely used methods for delineating individual exposure space based on one subject’s GPS trajectory data.

To compare the proposed analytical framework for food environment exposure measurement with other methods, four widely used exposure assessment methods [52,81,82,83,84,85,86] were also implemented with the same dataset. The four methods are GPS trajectory buffers (GTBs), standard deviation ellipses with one or two standard deviation(s) (SDE1, SDE2) and minimum convex polygons (MCPs). Figure 8 illustrates these four methods based on one subject’s GPS trajectory (note that the geographic coordinates of the GPS tracks shown in this figure have been modified for the purpose of human subjects’ protection). The GTBs were created by a 100-meter 2-D buffer along the participant’s GPS trajectories. The buffering area covered all the daily activity locations that this subject visited in the study period. The SDE is another widely-used method for exposure space delineation. Based on the transformed mean center and the rotated major and minor axes of all the GPS points of the subject, an ellipse was obtained based on either one or two standard deviation(s) of the distances between all pairs of GPS points. The SDEs represent the spatial distribution and directional trends of the subject’s activity locations and normally does not include all of the GPS points. The MCP is the smallest convex polygon that contains all the GPS points of the subject, which covers all the daily activity locations. With the same data of BMI-unhealthy food outlets, exposure to BMI-unhealthy food environment was calculated as the density of BMI-unhealthy food outlets in these four delineations of exposure space. The results were standardized with z-score transformation and compared with those obtained using the ECCs and ECEIs. 

### 2.7. Analytical Approach

To compare the performance of the environmental context cubes (ECCs) with different distance-decay methods at various spatial and temporal resolutions, as well as the exposure assessment results between the environmental context exposure index (ECEI) and other widely used food environment exposure measurements, we examined all these measurements and their relationship with participants’ overweight status using binary logistic regression models, which are widely used in public health studies [44,46,87]. The response variable is individual overweight status (0: non-overweight; 1: overweight or obese) based on participants’ BMI (non-overweight: BMI < 25.0 kg/m^2^; overweight or obese: BMI ≥ 25.0 kg/m^2^), while the independent variable is individual food environment exposure. The models were controlled for subjects’ age, gender, and education level. A total of 22 models were built with different measurements of BMI-unhealthy food environment exposure (18 measured by the ECEI based on various ECCs and 4 measured by other methods). The performance of these models was compared by the Akaike information criterion (AIC), Nagelkerke R^2^, likelihood ratio chi-square (LR χ^2^) and the corresponding *p*-value, which indicate the robustness of the model. The more robust a model is, the better exposure assessment will be obtained. In addition, the models were compared to see if a significant association existed between individual food environment exposure and overweight status. 

## 3. Results

### 3.1. Variation in Food Environment Exposure Measurements with Different Methods

**Figure 9:**
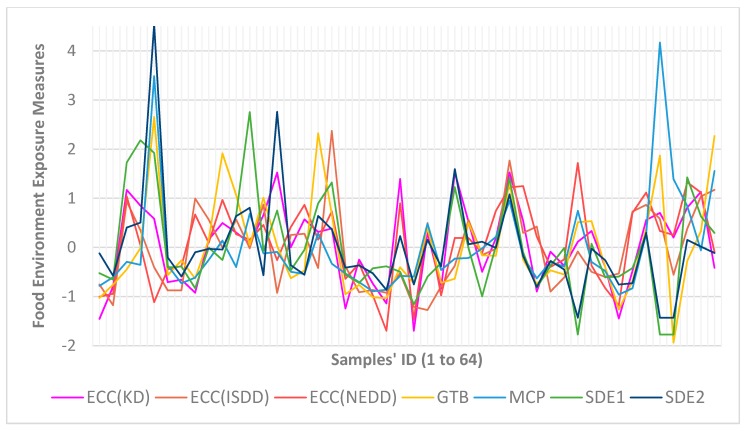
Comparison of the exposure measures obtained with different methods for each participant. (ECC: environmental context cube; KD: kernel density estimation; ISDD: inverse-square distance decay function; NEDD: negative-exponent distance decay function; GTB: GPS trajectory buffers; MCP: minimum convex polygons; SDE1: standard deviation ellipses with one standard deviation; SDE2: standard deviation ellipses with two standard deviations.)

Individual food environment exposures were measured by the environmental context cubes (ECCs) and the environmental context exposure indexes (ECEIs) using three different distance-decay methods, three different spatial resolutions, and two different temporal resolutions, as well as other four widely used methods (GTB, MCP, SDE1, and SDE2). Figure 9 illustrates the standardized measures of these methods for each participant. In the figure, exposures measured by the ECCs with three distance-decay methods include only those with the highest spatial (100 m × 100 m) and temporal resolution (10 min), since they captured the finest detail of the spatial and temporal dynamics of the food environment. The measurement results of the ECCs with different spatial and temporal resolutions will be compared and discussed in the following sections. The horizontal axis of Figure 9 indicates the 46 participants in the study, while the vertical axis shows the food environment exposure measures. The figure indicates that different methods give considerably different exposure measures for the same participant. 

To investigate the relationship among all the exposure measures obtained using different methods, we perform bivariate Pearson correlation analysis between each pair of the measures. Table A1 illustrates the results, which indicates that more than half of the pairs do not have significant correlations, including the pairs of ECC(KD)–MCP, ECC(ISDD)–MCP, ECC(ISDD)–SDE1, ECC(ISDD)–SDE2, ECC(NEDD)–GTB, ECC(NEDD)–MCP, ECC(NEDD)–SDE1, ECC(NEDD)–SDE2, GTB–SDE1, MCP–SDE1 and MCP–SDE2. Although the other pairs show significant correlations, most of the coefficients are smaller than 0.6, which indicate moderate to low associations. Only the pairs ECC(KD)–ECC(NEDD), ECC(ISDD)–ECC(NEDD), GTB–MCP and SDE1–SDE2 shows strong associations. It is reasonable that the results of the ECCs with different distance-decay methods are correlated with each other since they share the same model and concepts, while SDE1 and SDE2 are also the same methods with various parameters. The results show that individual exposures measured by different methods are mostly different from and not correlated with each other, which indicates the existence of the UGCoP. Therefore, it is worth comparing these measurements and exploring the accurate ways to assess individual food environment exposures.

### 3.2. Comparing the Performance of Food Environment Exposure Measurement Methods 

Table A2 shows the results of the binary logistic regression models with different measurements of individual BMI-unhealthy food environment exposure on the relationships between food environment exposure and overweight status. In the table, models KD100T10, KD100T30, KD150T10, KD150T30, KD200T10, and KD200T30 use exposures measured based on ECCs with KD as the distance-decay function in different spatial and temporal resolutions. In addition, models ISDD100T10, ISDD100T30, ISDD150T10, ISDD150T30, ISDD200T10, and ISDD200T30 use the exposure assessed based on ECCs with ISDD in various spatial and temporal resolutions. Furthermore, models NEDD100T10, NEDD100T30, NEDD150T10, NEDD150T30, NEDD200T10, and NEDD200T30 use exposure evaluated based on ECCs with NEDD in different spatial and temporal resolutions. Lastly, models M-GTB, M-MCP, M-SDE1, M-SDE2 use the measurement of individual food environment exposure based on four widely used methods (GTB, MCP, SDE1, and SDE2). The table shows that all the logistic regression models are statistically significant with *p*-value < 0.001. The models explained at least 45% (Nagelkerke R^2^) of the variance of participants’ overweight status. Among these models, the most robust one is the ISDD100T10 with the smallest AIC (45.552), largest LR χ^2^ (28.217), and *p*-value < 0.001. The model explained 61.13% (Nagelkerke R^2^) of the variance of participants’ overweight status. The least robust model is the KD150T10 (AIC = 54.240, LR χ^2^ = 19.529), which only explained 46.12% of the variance.

Among the ECC models, the food environment exposures estimated by ISDD generates the best results, while the ones estimated by KD generate the worst results. Considering various spatial and temporal resolutions of the ECC, the finer the resolution, the better the results. For instance, among the ECC models with specific distance-decay function, the most robust model is always the one with the finest spatial resolution: KD100T30 (AIC = 53.879, Nagelkerke R^2^ = 0.4681, LR χ^2^ = 19.891) for ECC(KD); ISDD100T10 (AIC = 45.552, Nagelkerke R^2^ = 0.6113, LR χ^2^ = 28.217) for ECC(ISDD); NEDD100T10 (AIC = 49.769, Nagelkerke R^2^ = 0.5420, LR χ^2^ = 24.001) for ECC(NEDD). In addition, the table indicates that ECCs with a temporal resolution of 10 min normally generate better results compared to the ones with a temporal resolution of 30 min with several exceptions.

Regarding the four widely used exposure assessment methods, GTB performs the best with AIC = 51.462, Nagelkerke R^2^ = 0.5124, LR χ^2^ = 22.308. However, the models with ECC(ISDD) and ECC(NEDD) still perform much better than the GTB-based model. It is worth noting that the models with ECC(KD) have the worst performance when compared to all the other methods, which may indicate that the effects of food outlets may not follow the decay patterns as depicted by a kernel density estimation.

### 3.3. Association between Food Environment Exposure and Overweight Status

The associations between food environment exposure based on different ECCs and participants’ overweight status are shown in Table A3. Almost all the models, except NEDD150T10, indicate that being female (compared to being male) is associated with higher odds of being overweight, while having a college degree and higher (compared high school degree and lower) is associated with lower odds of being overweight. However, significant association between BMI-unhealthy food environment exposure and overweight status is observed for only three of the models (ISDD100T10, ISDD100T30, and NEDD100T10). Higher unhealthy food environment exposure is found to be significantly associated with higher odds of being overweight in models ISDD100T10 (odds ratio (OR): 6.81; 95% confidential interval (CI): 1.76, 45.3; *p*-value < 0.01), ISDD100T30 (OR: 4.35; CI: 1.27, 22.62; *p*-value < 0.1) and NEDD100T10 (OR: 3.13; CI: 1.08, 11.47; *p*-value < 0.1). Referring to the performance of models discussed above, these three models are also the most robust models with the lowest AIC and highest LR χ^2^, as well as the highest explanation rate of the variance in participants’ overweight status. 

Table A4 lists the results of the binary logistic regression models based on the other four widely used methods. The models M-GTP, M-MCP and M-SDE1 indicate that being female is associated with higher odds of being overweight while having a college degree and higher is associated with lower odds of being overweight. M-SDE2 is the only model that does not find an association between education level and overweight status. Interestingly, all these four models did not find any significant association between BMI-unhealthy food environment exposure and participants’ overweight status.

The results indicate that the proposed framework generates better measurements of individual food environment exposures when compared to other widely used methods. This suggests the inconsistent findings in previous studies may be partly due to the methods used. Significant associations between BMI-unhealthy food environment exposures and overweight status were found in the three most robust models (ISDD100T10, ISDD100T30, and NEDD100T10). Being the most robust model, ISDD100T10 (explained 61.13% of the variance in participants’ overweight status) found that higher unhealthy food environment exposure (OR: 6.81; CI: 1.76, 45.3; *p*-value < 0.01) is significantly associated with higher odds of being overweight. 

## 4. Discussion

The proposed framework generated more reasonable and reliable results when compared to other methods, and thus obtained more accurate individual food environment exposures assessment. Regarding the distance-decay methods for generating the ECC, the ISDD represents the dynamic environmental contexts more accurately, and the ECC(ISDD) with a spatial resolution of 100 m × 100 m and a temporal resolution of 10 min performs best with the most robust regression models. Regarding the spatial and temporal resolution of the ECC, the finer the spatial and temporal resolution, the better the performance of the model. This suggests the existence of scale effects when using the ECC for measuring individual exposures. Thus, future application of the ECC may need to consider proper spatial and temporal resolution in order to generate reliable results.

The framework proposed in this study can help to mitigate the UGCoP. With respect to the spatial dimension, contextual units or areas in the study were not based on arbitrary pre-defined spatial boundaries (e.g., census tracts) but were delineated by ISTTs based on participants’ actual movement trajectories (GPS tracks). This is significantly different from the methods used in most previous studies, which tended to measure contextual influences based on static residential neighborhoods that may not accurately represent the actual areas that exert contextual influences on individual behavior or health outcomes [59,88,89,90]. On the other hand, temporal variations in relevant environmental contexts were handled dynamically: The influence of the food outlets was measured with consideration of their business hours in order to more accurately capture their contextual effects. Taking into account the complex spatial and temporal configuration of individual contextual exposure, the proposed framework and methods help to mitigate UGCoP.

The ECEI based on the ECC and ISTT in the study is a quantitative measure of individual exposure to environmental contexts in unit time, which may be used as a standard measure of individual contextual exposure. It provides a useful standardized tool for environmental exposure assessment, while capturing the spatial and temporal variations in environmental contexts. It is also flexible to implement the ECEI for different research questions concerning different environmental contexts using the two user-defined parameters $B_{r}$ and $W_{i}$. These two parameters can be explored in further studies to fit the research question. The index may be further used for comparative analysis of environmental exposures between different individuals or groups. In addition, the ECEI may be utilized to examine the relationships between environmental contexts and other health outcomes. The observed association may be used to investigate and identify high-risk environmental contexts and provide decision support for policy-making in public health.

Lastly, this research has several limitations that need to be addressed in future studies. First, this study implemented the proposed framework based on a relatively small sample of participants who live near parks. Larger GPS datasets with more subjects from different study sites are thus needed in future research to further evaluate the robustness of the framework. Second, this study only applied the framework to food environment exposures; further studies are needed to assess its effectiveness for addressing other health issues, such as physical activity and mental health. Third, we implemented the three distance-decay methods in Euclidean distance without considering transport modes and the configuration of road networks. More sophisticated methods [91] that incorporate transport modes in the ECC would help to further the application of the framework and has significant potential to better represent the environmental context. We will develop the ECC along this line in future studies. Fourth, since there is no data on participants’ actual activities, there may be some uncertainty in the exposure measure. For instance, working and eating at a fast-food restaurant may mean different exposure and have different effects on a participant’s body weight. If activity diary data are available, activity types can be integrated into the calculation of the ECEI by differentiating the contextual effects of different types of activity. Fifth, the proposed methodology can only explore the association between environmental exposures and health outcomes. Further investigations (e.g., controlled experiments or longitudinal studies) are still needed to validate any causal relationships. 

## 5. Conclusions

This study developed and implemented an analytical framework to dynamically represent food environment and derive individual environmental exposures that effectively integrates human movement in space and time (e.g., GPS trajectories). The proposed framework incorporates the dynamics of the food environment into the environmental context cube (ECC), captures individual exposure space with the individual space-time tunnel (ISTT), and assesses the effects of individual exposure on people’s overweight status with the environmental context exposure index (ECEI). The framework was designed to examine individual food environment exposure but can also be used in a wide range of environmental health studies.

## Figures and Tables

**Table 1:** The socio-demographic characteristics of the participants in this study.

Socio-Demographic Variables		Percentage
Gender	Male	39.13%
	Female	60.87%
Age (years old)	18–30	56.52%
	31–65	41.30%
	65+	2.18%
Education	With College Degree or Higher (≥College Degree)	56.52%
	With High School Degree or Lower (<College Degree)	43.48%
OverweightStatus	Overweight	50%
	Non-overweight	50%

## Appendix A

**Table A1:** The results of the bivariate Pearson correlation analysis between each pair of the methods for food environment exposure assessment.

Methods	ECC(KD)	ECC(ISDD)	ECC(NEDD)	GTB	MCP	SDE1	SDE2
ECC(KD)	-	0.407 *	0.658 *	0.438 *	0.364	0.508 *	0.476 *
ECC(ISDD)	-	-	0.642 *	0.396 *	0.198	0.358	0.057
ECC(NEDD)	-	-	-	0.269	0.180	0.168	−0.033
GTB	-	-	-	-	0.629 *	0.345	0.439 *
MCP	-	-	-	-	-	0.099	0.291
SDE1	-	-	-	-	-	-	0.699 *
SDE2	-	-	-	-	-	-	-

* *p*-value < 0.05.

**Table A2:** The results of binary logistic regression models with different measurements of individual BMI-unhealthy food environment exposure for relationships between food environment exposure and overweight status.

Model ^a,b^	Method	Spatial Resolution	Temporal Resolution	AIC	Nagelkerke *R*^2^	LR χ^2^	*p*-Value
KD100T10	ECC (KD)	100 m × 100 m	10 min	53.898	0.4677	19.872	0.00053 ***
KD100T30			30 min	53.879	0.4681	19.891	0.00052 ***
KD150T10		150 m × 150 m	10 min	54.240	0.4612	19.529	0.00062 ***
KD150T30			30 min	54.228	0.4615	19.542	0.00061 ***
KD200T10		200 m × 200 m	10 min	54.198	0.4620	19.571	0.00061 ***
KD200T30			30 min	54.223	0.4616	19.546	0.00061 ***
ISDD100T10	ECC (ISDD)	100 m × 100 m	10 min	45.552	0.6113	28.217	0.00001 ***
ISDD100T30			30 min	48.567	0.5624	25.202	0.00005 ***
ISDD150T10		150 m × 150 m	10 min	53.272	0.4794	20.498	0.00040 ***
ISDD150T30			30 min	52.801	0.4881	20.969	0.00032 ***
ISDD200T10		200 m × 200 m	10 min	53.124	0.4822	20.645	0.00037 ***
ISDD200T30			30 min	54.073	0.4644	19.696	0.00057 ***
NEDD100T10	ECC (NEDD)	100 m × 100 m	10 min	49.769	0.5420	24.001	0.00008 ***
NEDD100T30			30 min	53.597	0.4734	20.172	0.00046 ***
NEDD150T10		150 m × 150 m	10 min	52.945	0.4855	20.825	0.00034 ***
NEDD150T30			30 min	51.792	0.5064	21.977	0.00020 ***
NEDD200T10		200 m × 200 m	10 min	54.132	0.4633	19.638	0.00059 ***
NEDD200T30			30 min	54.199	0.4650	19.571	0.00061 ***
M-GTB	GTB	-	-	51.462	0.5124	22.308	0.00017 ***
M-MCP	MCP	-	-	52.390	0.4956	21.380	0.00027 ***
M-SDE1	SDE1	-	-	53.542	0.4744	20.228	0.00045 ***
M-SDE2	SDE2	-	-	51.753	0.5071	22.016	0.00020 ***

^a^ N = 46 subjects, ^b^ Response variable: 0 non-overweight; 1 overweight, *** *p*-value < 0.001, AIC: Akaike information criterion (the smaller the value, the better the model fit), LR χ^2^: likelihood ratio chi-square (the larger the value, the better the model fit).

**Table A3:** The association between food environment exposure and overweight status analyzed by binary logistic regression models with food environment exposure measured by different ECCs.

Variables	Model ^a,b^	β (95% CI)	OR (95% CI)	Model ^a,b^	β (95% CI)	OR (95%)
Gender (Female)	KD100T10	2.56 *** (0.97, 4.63)	12.97 *** (2.64, 102.57)	KD100T30	2.57 ** (0.97, 4.63)	13.01 ** (2.65, 102.77)
Age		0.05 (−0.02, 0.14)	1.05 (0.98, 1.14)		0.05 (−0.02, 0.14)	1.05 (0.98, 1.15)
Education (≥College Degree)		−2.37 ** ( −4.46, −0.72)	0.09 ** (0.01, 0.49)		−2.37 ** (−4.47, −0.73)	0.09 ** (0.01, 0.48)
Env. Exp.		0.28 (−0.64, 1.27)	0.09 (0.01, 0.49)		0.29 (−0.64, 1.28)	1.34 (0.53, 3.60)
Gender (Female)	KD150T10	2.53 *** (0.91, 4.63)	12.62 *** (2.49, 102.72)	KD150T30	2.54 ** (0.92, 4.64)	12.73 ** (2.51, 103.68)
Age		0.05 (−0.02, 0.14)	1.05 (0.98, 1.15)		0.05 (−0.02, 0.14)	1.05 (0.98, 1.14)
Education (≥College Degree)		−2.43 *** (−4.56, −0.76)	0.09 *** (0.01, 0.47)		−2.42 ** (−4.55, −0.76)	0.09 ** (0.01, 0.47)
Env. Exp.		0.06 (−0.94, 1.04)	1.06 (0.39, 2.82)		0.08 (−0.90, 1.04)	1.08 (0.41, 2.83)
Gender (Female)	KD200T10	2.54 *** (0.94 4.62)	12.68 *** (2.56, 101.55)	KD200T30	2.53 ** (0.94, 4.61	12.57 ** (2.56, 100.23)
Age		0.05 (−0.02, 0.13)	1.05 (0.98, 1.14)		0.05 (−0.02, 0.14)	1.05 (0.98, 1.14)
Education (≥College Degree)		−2.44 *** (−4.53, −0.83)	0.09 *** (0.01, 0.44)		−2.45 ** (−4.53, −0.83)	0.09 ** (0.01, 0.43)
Env. Exp.		0.11 (−0.84, 1.02)	1.12 (0.43, 2.77)		0.08 (−0.87, 0.99)	1.09 (0.42, 2.69)
Gender (Female)	ISDD100T10	3.61 *** (1.58, 6.37)	36.83 *** (4.87, 584.49)	ISDD100T30	3.30 ** (1.41, 5.84)	27.13 ** (4.10, 342.79)
Age		0.03 (−0.04, 0.12)	1.04 (0.96, 1.13)		0.03 (−0.05, 0.12)	1.03 (0.95, 1.12)
Education (≥College Degree)		−2.13 ** (−4.42, −0.31)	0.12 ** (0.01, 0.74)		−1.93 * (−4.09, −0.19)	0.14 * (0.02, 0.83)
Env. Exp.		1.92 ** (0.57, 3.81)	6.81 ** (1.76, 45.3)		1.47 * (0.24, 3.12)	4.35 * (1.27, 22.62)
Gender (Female)	ISDD150T10	2.90 *** (1.12, 5.23)	18.14 *** (3.06, 186.21)	ISDD150T30	2.92 ** (1.17, 5.21)	18.48 ** (3.21, 182.24)
Age		0.04 (−0.03, 0.13)	1.04 (0.97, 1.14)		0.04 (−0.03, 0.13)	1.05 (0.97, 1.14)
Education (≥College Degree)		−2.34 ** (−4.42, −0.71)	0.10 ** (0.01, 0.49)		−2.36 * (−4.45, −0.72)	0.09 * (0.01, 0.48)
Env. Exp.		0.45 (−0.44, 1.42)	1.57 (0.64, 4.12)		0.51 (−0.31, 1.47)	1.67 (0.73, 4.36)
Gender (Female)	ISDD200T10	2.68 *** (1.03, 4.86)	14.61 *** (2.80, 129.08)	ISDD200T30	2.57 ** (0.96, 4.68)	13.09 ** (2.62, 107.41)
Age		0.05 (−0.02, 0.13)	1.05 (0.98, 1.14)		0.05 (−0.02, 0.13)	1.05 (0.98, 1.14)
Education (≥College Degree)		−2.60 *** (−4.77, −0.94)	0.07 *** (0.01, 0.39)		−2.47 ** (−4.55, −0.85)	0.09 ** (0.01, 0.43)
Env. Exp.		0.49 (−0.41, 1.46)	1.63 (0.66, 4.31)		0.19 (−0.69, 1.11)	1.21 (0.50, 3.04)
Gender (Female)	NEDD100T10	2.81 *** (1.10, 5.04)	16.55 *** (3.02, 154.12)	NEDD100T30	2.57 ** (0.98, 4.64)	13.07 ** (2.66, 103.57)
Age		0.05 (−0.03, 0.13)	1.05 (0.97, 1.14)		0.05 (−0.02, 0.13)	1.05 (0.98, 1.14)
Education (≥College Degree)		−1.98 ** (−4.13, −0.26)	0.14 ** (0.02, 0.77)		−2.20 * (−4.35, +0.49)	0.11 * (0.01, 0.61)
Env. Exp.		1.14 * (0.08, 2.44)	3.13 * (1.08, 11.47)		0.37 (−0.51, 1.40)	1.44 (0.60, 4.06)
Gender (Female)	NEDD150T10	2.90 *** (1.15, 5.19)	18.25 *** (3.17, 180.20)	NEDD150T30	3.00 ** (1.25, 5.29)	20.09 ** (3.49, 199.09)
Age		0.04 (−0.04, 0.12)	1.04 (0.96, 1.13)		0.04 (−0.04, 0.12)	1.04 (0.96, 1.13)
Education (≥College Degree)		−2.19 (−2.29, −0.52)	0.11 (0.01, 0.59)		−2.21 * (−4.31, −0.55)	0.11 * (0.01, 0.58)
Env. Exp.		0.57 (−0.40, 1.62)	1.76 (0.67, 5.06)		0.72 (−0.17, 1.77)	2.06 (0.84, 5.81)
Gender (Female)	NEDD200T10	2.53 *** (0.94, 4.62)	12.64 *** (2.57, 101.77)	NEDD200T30	2.50 ** (0.92, 4.57)	12.22 ** (2.51, 96.49)
Age		0.05 (−0.02, 0.13)	1.05 (0.98, 1.14)		0.05 (−0.02, 0.14)	1.06 (0.98, 1.15)
Education (≥College Degree)		−2.46 *** (−4.54, −0.85)	0.09 *** (0.01, 0.43)		−2.48 ** (−4.57, −0.86)	0.08 ** (0.01, 0.42)
Env. Exp.		0.14 (−0.68, 0.93)	1.15 (0.51, 2.54)		−0.09 (−0.89, 0.67)	0.91 (0.41, 1.95)

^a^ N = 46 subjects, ^b^ Response variable: 0 non-overweight; 1 overweight, * *p*-value < 0.1, ** *p*-value < 0.01, *** *p*-value < 0.001, Gender: 0-male, 1-female, Education: 0-without a college degree or higher; 1-with a college degree or higher, Env. Exp.: BMI-unhealthy food environment exposure measured by a specific method.

**Table A4:** The association between food environment exposure and overweight status analyzed by the binary logistic regression models with the food environment exposure measured by the other four widely used methods.

Variables.	Model ^a,b^	β (95% CI)	OR (95% CI)
Gender (Female)	M-GTP	3.12 *** (1.29, 5.62)	22.68 *** (3.62, 275.66)
Age		0.08 (0.00, 0.17)	1.08 (10.00, 1.19)
Education (≥College Degree)		−2.48 ** (−4.72, −0.76)	0.08 ** (8.91, 0.47)
Env. Exp.		0.29 (−0.05, 0.69)	1.34 (9.52, 2.00)
Gender (Female)	M-MCP	2.57 *** (0.95, 4.70)	13.10 *** (2.60, 109.75)
Age		0.06 (−0.01, 0.15)	1.06 (0.99, 1.17)
Education (≥College Degree)		−2.53 *** (−4.70, −0.86)	0.08 *** (0.01, 0.42)
Env. Exp.		0.56 (−0.23, 1.53)	1.74 (0.79, 4.60)
Gender (Female)	M-SDE1	2.32 ** (0.66, 4.43)	10.16 ** (1.93, 83.83)
Age		0.06 (−0.02, 0.14)	1.06 (0.98, 1.15)
Education (≥College Degree)		−2.74 *** (−5.03, −0.99)	0.06 *** (0.01, 0.37)
Env. Exp.		−0.24 (−0.90, 0.31)	0.78 (0.40, 1.36)
Gender (Female)	M-SDE2	2.33 ** (0.64, 4.51)	10.26 ** (1.90, 90.62)
Age		0.04 (−0.03, 0.13)	1.05 (0.97, 1.14)
Education (≥College Degree)		−2.83 (−5.17, −1.07)	0.06 (0.01, 0.34)
Env. Exp.		−0.57 (−1.36, 0.12)	0.57 (0.26, 1.12)

^a^ N = 46 subjects, ^b^ Response variable: 0 non-overweight; 1 overweight, ** *p*-value < 0.01, *** *p*-value < 0.001.
